# Mapping Variability in Bovine Respiratory Disease Risk Factors in Beef Production Systems: A Scoping Review

**DOI:** 10.3390/ani16111726

**Published:** 2026-06-04

**Authors:** Adeolu Adekunle, Alexcia Gaines, Natalie Estefano, Devyani Lenin, Piyush Hole, Rhythm Khandelwal, Reinaldo Cooke, Karun Kaniyamattam

**Affiliations:** 1 Department of Animal Science, Texas A&M University, College Station, TX 77843, USA; 2 College of Veterinary Medicine and Biomedical Sciences, Texas A&M University, College Station, TX 77843, USA; 3 Department of Mathematics, Texas A&M University, College Station, TX 77843, USA; 4 College of Engineering, Texas A&M University, College Station, TX 77843, USA

**Keywords:** bovine respiratory disease, epidemiology, beef cattle, disease management, beef production system, risk factors

## Abstract

This scoping review examines bovine respiratory disease (BRD) risk factors across beef production systems to clarify their contribution to disease occurrence and outcomes and to identify gaps that limit effective prevention. Following the PRISMA-ScR guidelines, eight databases (EBSCOhost, Google Scholar, MDPI, PubMed, ScienceDirect, Journal of Animal Science, Journal of Dairy Science, and Web of Science) were searched for peer-reviewed studies published between 2004 and 2024, yielding 91 eligible studies from 133 screened articles. Data were charted to assess how BRD risk factors have been investigated across cow–calf, stocker/backgrounding, and feedlot systems and categorized into biological, operational, and environmental domains. Twenty-three major risk factors were identified, with transportation stress and commingling most frequently studied in stocker and feedlot systems, while management-related factors such as vaccination were more commonly emphasized in cow–calf operations. The evidence base was heavily skewed toward feedlot populations (62%), with limited focus on cow–calf (13%) and stocker (7%) systems and few longitudinal studies spanning multiple production stages. Overall, the findings reveal substantial heterogeneity in research emphasis and highlight critical gaps in early-life and cross-stage risk assessment. These findings underscore an urgent need for longitudinal, cross-stage cohort studies and standardized BRD surveillance frameworks to address the early-life evidence gap and provide a foundation for more effective, data-driven, integrated disease prevention strategies.

## Simple Summary

Bovine respiratory disease is one of the most common and costly illnesses affecting cattle raised for beef, yet its causes can vary depending on how and where animals are raised. This study reviewed existing research to better understand the main factors that increase the risk of this disease across different stages of beef production. We analyzed 91 studies published over the past two decades and grouped risk factors into three areas: the animal itself, how it is managed, and its environment. We found that stress from transportation and mixing animals from different sources are frequently linked to disease in later production stages, while early-life management practices, such as vaccination, are more often studied in younger animals. However, most research focuses on cattle in feedlots, with far less attention given to earlier stages of life. This gap limits our ability to fully prevent disease before it develops. Overall, our findings highlight the need for more balanced research across all stages of production to improve animal health, reduce economic losses, and support more sustainable beef production systems.

## 1. Introduction

Bovine respiratory disease (BRD) is the costliest disease affecting beef cattle production due to its adverse effects on cattle performance, associated economic costs, and treatment methods [1]. This disease has a multifactorial etiology, resulting from various bacterial and viral pathogens [2,3], and causes numerous chronic disorders, such as pneumonia in cattle [4]. The complexity of BRD’s onset makes managing and preventing this disease in large integrated beef production systems difficult. Several prior reviews have addressed components of this problem: Ref. [5] examined BRD risk factors in cow–calf systems but were limited in cross-stage comparison; Ref. [6] covered epidemiological evidence for risk factors but did not map research frequency across production stages. No prior scoping review has comprehensively compared BRD risk factors across all three production stages using a structured, frequency-based approach. Accordingly, this scoping review systematically maps the distribution of research attention devoted to BRD risk factors across beef production systems, classifies these risk factors by biological, environmental, and operational domains, and identifies critical gaps that constrain effective, stage-specific disease management.

Integrated U.S. beef production comprises three main stages: the cow–calf stage, in which breeding cows produce and raise calves until weaning (typically 6–8 months of age); the stocker or backgrounding stage, in which weaned calves are grown on pasture or in dry lots to gain weight before feedlot entry; and the feedlot stage, where cattle are fed high-energy diets to reach optimal market weight [7]. BRD is prevalent across all stages of beef production; however, its burden is most pronounced during the post-weaning transitional stage [8] when freshly weaned calves are transported and commingled at stocker and feedlot facilities. Despite the emphasis on BRD in the feedlot, it is seen in all stages of beef production, including cow–calf and stocker operations. For example, BRD also accounts for about 15.9% of all cattle and 23% of all calf deaths in cow–calf operations [8,9]. The risk factors and onset of BRD at different stages of beef production appear different due to differing host factors and housing environments associated with each stage, making a universal, sustainable method of BRD management largely unfeasible. A study assessing the impacts of BRD on performance at an 18% morbidity rate and a 2.1% mortality rate estimated an average net loss of AUD$1647.53 (USD$1136.80 at 0.69 AUD/USD year 2020 average exchange rate) per mortality [1]. Adjusted for inflation, the current equivalent loss per mortality is likely substantially higher [10]. While clinical BRD cases produced the worst performance among cattle, even subclinical BRD cases and those that recovered through treatment showed decreased carcass weights [10] and returned a lower than ideal profit at slaughter [1].

The implications of BRD go beyond temporary ailments. BRD often leads to chronic health issues and poses a threat to animal welfare in the beef production system [11]. It also causes an overall decrease in the productivity of individual cattle and the efficiency of the production system [10], as the need for new protocols and treatment is constantly increasing [12]. The cost of these procedures, as well as the lost profit from cattle that either do not make it to market due to BRD or have decreased carcass value from BRD, all add up to a decrease in the overall profitability of feedlots. Average BRD treatment costs around $23.60 per head, cumulating to an excess spending of $75 million annually on BRD treatment, and this cost only continues to rise [8]. These implications corroborate the need for further research on risk factors at each stage of integrated beef cattle production to develop improved management protocols, treatments, and preventive measures to mitigate the effects of BRD across all levels of production.

### 1.1. Causes and Clinical Signs

BRD is shaped by dynamic interactions among pathogen, host, and environmental factors [6,13,14]. Risk factors contributing to stress and genetic susceptibility also influence the pathogenesis of BRD [15]. Onset of BRD typically follows a multi-step pathogenesis in which primary viral infection disrupts mucosal integrity and induces transient immunosuppression, which in turn predisposes the lower respiratory tract to colonization by opportunistic bacterial pathogens, ultimately resulting in bacterial bronchopneumonia [6,16,17]. Commonly isolated bacteria belong to the Pasteurellaceae and Mycoplasmataceae families, while notable viruses include bovine respiratory syncytial virus (BRSV), bovine coronavirus (BcoV), bovine herpesvirus (BHV), and bovine viral diarrhea virus (BVDV) [2]. *Mycoplasma bovis* is one of the most widespread bacteria associated with BRD and contributes to the greatest morbidity [3]. Occasionally, fungi belonging to the *Aspergillus* genus and “lungworm” parasites are known to trigger BRD [2]. These varying causes can make the proper treatment of BRD difficult to pinpoint, which is why blanket vaccination [18] and indiscriminate metaphylaxis (or antimicrobial use) [19] can sometimes yield unintended consequences, doing more harm than good by increasing the risk of pathogen resistance without guaranteeing prevention of BRD. Host-related risk factors that predispose cattle to BRD include poor immune status, young age, low body weight, and the general low lung capacity of ruminants [20]. Hereford cattle were found to be 10 times more likely to undergo BRD treatment, signifying that the breed of cattle significantly affects susceptibility [21].

Subtle clinical signs during the onset of BRD include mild depression and lethargy, slow and purposeless movements, anorexia, hypodipsia, slightly bowed heads, ocular and nasal secretions, and frequent nostril cleaning using the tongue [4,22,23]. Clinical signs that signify more advanced BRD infection can present as severe depression, heavy or labored breathing, sunken eyes, intermittent cough, fever (>40 °C), and purulent nasal discharge [24]. Scoring systems such as the DART (depression, appetite, respiration, and temperature) method are often used to identify BRD [25]. Relying on visualizing clinical signs to diagnose BRD becomes troublesome in cases where infected individuals are asymptomatic, clinical signs are subtle, or are identified “too late”.

### 1.2. Treatment and Prevention

Treatment methods for BRD vary depending on the pathogen causing the infection, but commonly include long-acting antimicrobials, antibacterials, nonsteroidal anti-inflammatory drugs (NSAIDs), and vaccinations. Vaccination against commonly isolated bacteria and viruses associated with BRD is widely used and accepted as an effective preventive measure, but it does not prevent BRD caused by other factors. Prevention methods for BRD often focus on minimizing risk factors within the environment and for cattle. Metaphylaxis is also a common practice among producers [10], with tetracyclines and macrolides among the antimicrobial agents commonly used, and the procedure calls for 7 days of oral administration [26].

Prevention encompasses a broad spectrum: vaccination programs [18,27], prophylactic and metaphylactic antimicrobial interventions [19], stress management practices during transportation and weaning, and biosecurity protocols. Minimizing risk factors that contribute to stress remains one of the most effective ways to prevent the occurrence and development of BRD. But it can also be the most challenging to implement. For example, a major risk factor contributing to the spread of BRD is commingling [6], in which cattle from different origins are introduced to each other, usually at the beginning of the stocker and feedlot phases of beef production. It is impractical to eliminate commingling in large feedlot operations where cattle are bought from multiple different stocking operations. Transportation is also a significant contributor to stress; however, providing adequate rest periods, food, and water during the process helps mitigate the strain on cattle [28], thereby reducing the risk of stress-induced BRD. Some risk factors exist before arrival at the feedlot and can be minimized through preconditioning, potentially lowering the chances of BRD infection [5].

Biosecurity measures represent foundational prevention strategies and should be regarded as a primary line of defense against BRD. Practices such as isolation of incoming cattle, sanitation of shared equipment, and restricted animal movement between facilities [10] reduce pathogen introduction and transmission independent of antimicrobial intervention [29]. The implementation of robust biosecurity protocols must be prioritized alongside—and in many contexts above—metaphylactic antimicrobial use, particularly given the global escalation of antimicrobial resistance (AMR) in livestock systems [18]. Novel preventive and treatment methods, including advanced sequencing techniques that support pathogen identification and inform vaccine development, and the application of artificial intelligence and machine learning (AI/ML) predictive algorithms, are continually being researched and developed [30,31,32].

### 1.3. Integrated Beef Production

**Figure 1:**
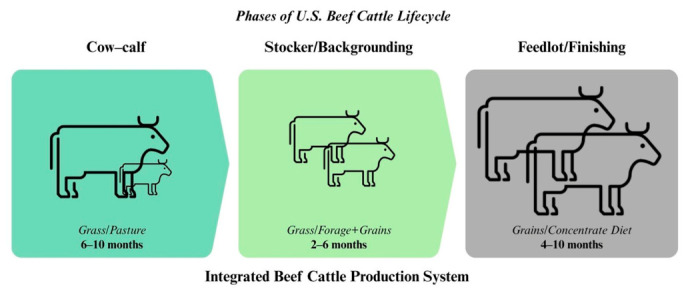
A diagrammatic representation of the three main stages of U.S. beef cattle production.

An integrated beef production system is a complex and dynamic supply chain encompassing a series of consecutive activities from breeding to processing [29] and consists of three main stages (Figure 1). The initial phase is the cow–calf operation, which raises calves, typically for up to a year after birth. Key management practices include ensuring adequate colostrum transfer for passive immunity, supported by robust husbandry and biosecurity protocols.

The transition from cow–calf operations is marked by weaning, a significant stressor that can negatively impact a calf’s immune system [5,33]. Multiple early-life risk factors compound this vulnerability: failure of passive immune transfer due to inadequate colostrum intake [34], early-life commingling with animals of diverse health backgrounds [35]; the immunological and physiological burden of long-distance transportation [36] and the physiological stress of weaning-induced maternal separation. These factors constitute the risk profile at the earliest stages of production. Following weaning, calves are often transported to auction markets, where commingling with cattle of diverse health backgrounds exacerbates stress and pathogen exposure. Subsequently, calves may enter stocker or backgrounding operations, where they are raised on pastures or in dry lots to achieve further weight gain before progressing to feedlots [37]. Maintaining health throughout these intermediate stages is paramount, as the foundational immune competence developed at the cow–calf level must be sustained across every sector of the production chain. Practices like preconditioning, which involve vaccinations and deworming, are designed to mitigate shipping and receiving stress, although they do not fully prevent illness [38]. Transportation itself is a major physiological stressor that can compromise immune responses, with long journeys and environmental conditions increasing the likelihood of co-infections and disease development [39]. Strategic management, such as avoiding immediate feedlot placement after saleyard exposure or mixing [40], can offer a protective effect against BRD.

The terminal phase of beef production involves feedlot operations, where cattle receive high-energy rations of forages and grains to reach their optimal slaughter weight. Entry into the feedlot is recognized as a high-risk period [39,41], with BRD being a primary concern for morbidity and mortality. Management at this stage focuses on optimizing feed intake, growth, and overall health outcomes, often utilizing advanced tools such as dynamic growth models [42] and production optimization tools [43,44] to predict individual animal performance and carcass characteristics. Ultimately, the value of beef is determined by factors like carcass weight, grade, and quality [45]. Achieving a sustainable integrated beef production system requires a comprehensive, multi-sector approach that addresses animal health and management throughout the animal’s entire lifetime and across all production sectors. This strategy is critical for mitigating economic losses, effectively controlling diseases, and meeting consumer demands, moving beyond fragmented, sector-specific efforts towards a unified industry-wide strategy.

### 1.4. Major Risk Factors of BRD

Risk factors for BRD can include environmental, biological, and operational factors [46]. Genetic factors can substantially influence cattle’s inherent susceptibility, which environmental factors can compound. Younger and lighter cattle are particularly susceptible to BRD due to their developing immune systems and the challenges of adapting to new environments [47]. An Australian feedlot study found breed, induction weight, and induction season to be strongly associated with BRD risk [41]. One major risk factor associated with operational practice is transportation stress, which can compromise the immune system and predispose cattle to respiratory infections. The presence of pathogens, specifically BCoV, BRSV, *M. bovis*, *Mannheimia haemolytica*, and *Pasteurella multocida*, was found to be significantly higher in a study of cattle undergoing transportation stress [36]. Additionally, the degree of commingling upon arrival at the feedlot heightens the risk of disease transmission. Environmental conditions, such as temperature fluctuations and humidity, further contribute to the incidence of BRD. Management practices, including the timing and administration of metaphylactic treatments, are also related to the risk of BRD. Inadequate or delayed interventions can lead to increased treatment failures [47]. Additional risk factors include herd size [48], the health or unknown status of purchased animals [25], and reproductive sourcing practices that affect herd biosecurity and may indirectly elevate BRD risk [10]. Practices across each stage of production often present overlapping risk factors; therefore, understanding and addressing these risk factors and their relationships is essential to managing and improving the health and welfare of cattle and combating BRD.

### 1.5. Gaps in Knowledge

Delving deeper into BRD’s prevalence and specific causes across the integrated beef system is vital for determining the scope of BRD and the next steps in managing the disease. With ongoing global sustainability concerns, the environment is constantly impacted, which, in return, impacts BRD’s emergence in new, evolving ways [49]. Despite extensive research on the various pathogens of BRD, there is still much to learn about the relationships between specific risk factors [50]. Pathogen resistance to antimicrobials is a growing issue that calls for the development of more efficient methods of controlling BRD [51]. This review aims to address two key research questions: (1) What is the research attention given to different risk factors of BRD across different stages of beef production? (2) What are the key interactions and overlaps between these different BRD risk factors?

## 2. Materials and Methods

### 2.1. PRISMA-ScR Protocol

The Preferred Reporting Items for Systematic reviews and Meta-Analyses extension for Scoping Reviews (PRISMA-ScR) 22-item checklist for scoping reviews [52] was used to ensure completeness and transparency of this review (Supplementary Table S1).

### 2.2. Eligibility Criteria (Inclusion and Exclusion)

This scoping review used a mixed-methods approach, including papers presenting quantitative and/or qualitative data.

Four inclusion criteria were applied: studies must include beef cattle operations (including beef on dairy); only peer-reviewed studies published within the last 20 years (2004–2024) were selected; all studies were written in or translated into English; all studies were conducted in, or evaluated, beef production in Australia, North America, and/or Europe. The screening process was conducted independently by at least three reviewers (A.G., D.L., and N.E.), with a fourth reviewer (A.A.) consulted to resolve disagreements. Disagreements were resolved through discussion; where consensus could not be reached, the article was included at the screening stage and re-evaluated at the full-text eligibility stage by a senior reviewer. The inter-reviewer disagreement rate was fewer than 5% of screened articles. A formal inter-rater reliability statistic (e.g., Cohen’s kappa) was not calculated and is acknowledged as a limitation in this study. Conference proceedings were included only if they presented original data meeting all other inclusion criteria. The inclusion of conference proceedings is acknowledged as a potential source of heterogeneity in evidence quality, and the weight of these sources is to be interpreted with appropriate caution.

### 2.3. Search Strategy

The search strategy aimed to be methodical, ensuring comprehensive coverage of relevant studies while minimizing bias. Key search terms included “BRD Risk Factors,” “BRD Predisposing Factors,” “Bovine Respiratory Disease” AND “Risk Factors,” “Beef Cattle Production,” “Cow-Calf,” “Stocker,” and “Feedlot.” Queries were run across eight databases: EBSCOhost, Google Scholar, MDPI, PubMed, ScienceDirect, Journal of Animal Science, Journal of Dairy Science, and Web of Science. Example Boolean search strings used: (“bovine respiratory disease” OR “BRD”) AND (“risk factor*” OR “predisposing factor*”) AND (“beef cattle” OR “feedlot” OR “cow-calf” OR “stocker”).

The 20-year search window (2004–2024) was selected to capture the period following the widespread adoption of integrated beef production, coinciding with three key contextual shifts: (1) the emergence of consolidated feedlot operations and changes in animal movement and commingling practices; (2) advances in molecular diagnostic and surveillance technologies that improved BRD pathogen detection; and (3) increasing regulatory and scientific attention to AMR in livestock, particularly following the 2006 EU ban on growth-promoting antibiotics and subsequent policy reforms in North America [29].

Duplicates were identified and removed using RefWorks reference management software prior to the screening stage, with a manual cross-check performed by two independent reviewers.

### 2.4. Data Extraction

Data extraction was conducted using a standardized data charting approach consistent with PRISMA-ScR guidance for scoping reviews [52], with the primary objective of mapping the breadth, focus, and distribution of BRD risk factors across integrated beef production systems. For each included study, key descriptive and analytical elements were extracted into a structured extraction table (Supplementary Table S2), including: author and year, study design, geographic region, production stage(s) examined (cow–calf, stocker/backgrounding, feedlot, or mixed), cattle population characteristics (when reported), key findings and all BRD-related risk factors were explicitly examined or discussed. Risk factors were recorded regardless of whether a statistically significant association with BRD was reported, consistent with the scoping objective of mapping research coverage and emphasis [53].

Risk factors were subsequently classified into three broad domains: biological (host-related), operational (management-related), and environmental. This classification framework was selected over alternative models (e.g., “host-pathogen-environment” or “endogenous-exogenous”) because it aligns with the management-oriented BRD literature [6,14], facilitates direct application to stage-specific prevention strategies, and accommodates the operational nuances central to livestock production systems. Overlaps among categories are acknowledged: for example, “stress” has both biological (physiological response) and operational (management-triggered) dimensions; classifications reflect the primary domain of each factor rather than exclusive categorization.

Quantitative data were not pooled or synthesized through meta-analytic techniques. Instead, quantitative information was used descriptively to document study-based productivity outcomes relevant to BRD and support interpretation of how frequently specific risk factors were examined across studies and production stages. Each included article contributed one unit to frequency counts regardless of study design, sample size, or whether the article was a primary study or a review article [53]. This approach reflects the scoping review objective of characterizing patterns of research focus and evidence availability, rather than weighing evidence by study size or estimating causal effects.

To avoid double-counting, risk factors extracted from review articles were recorded as novel insights only if explicitly proposed by the review authors as original synthesis conclusions not traceable to a specific cited primary study within the same review. Risk factors that review articles were attributed to, including primary studies, were not re-counted; instead, they confirmed coverage within the primary literature. This distinction was operationalized by recording the source type (primary vs. review) and flagging any risk factor mentioned exclusively in review articles for conservative interpretation.

This data extraction strategy was designed to align with the primary aims of this scoping review: to map the landscape of BRD risk factor research across beef production systems, identify areas of concentrated evidence, and highlight gaps for future investigation.

## 3. Data Analysis

**Figure 2:**
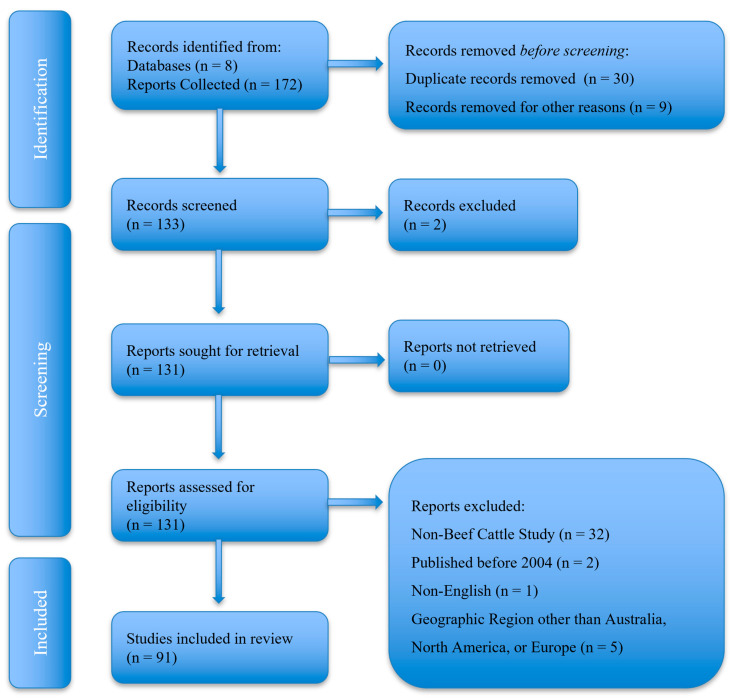
A PRISMA flow diagram demonstrating the screening process. Of the 172 reports collected, 39 had to be removed due to being duplicates or for other reasons. Of the remaining 133 reports, 2 were excluded for not relating to the topic. From there, 40 reports were excluded using the inclusion criteria established previously. In the end, 91 studies were included in this review.

The pre-screening process removed 39 studies that focused outside of BRD, were duplicate studies, or did not include the key search terms. This removal process followed an identification stage in which 172 studies were selected as potentially relevant to BRD. A total of 133 studies were screened thereafter, representing only those that met the initial relevance-based pre-screening. Two studies were removed during screening for failing to focus on risk factors. In total, 131 studies were retrieved after screening. These studies were then assessed for eligibility using the four criteria detailed previously. Of these papers, 5 were excluded specifically for being conducted outside of the target regions, 32 for focusing on “non-beef” studies, 2 for being published before 2004, and 1 research paper not written in English. In the end, 91 studies were included in the review. The entire screening process is demonstrated in the diagram below (Figure 2).

## 4. Results and Discussion

### 4.1. Risk Factors Studied

The 91 [5,6,9,12,13,14,15,21,27,34,35,36,37,41,47,48,54,55,56,57,58,59,60,61,62,63,64,65,66,67,68,69,70,71,72,73,74,75,76,77,78,79,80,81,82,83,84,85,86,87,88,89,90,91,92,93,94,95,96,97,98,99,100,101,102,103,104,105,106,107,108,109,110,111,112,113,114,115,116,117,118,119,120,121,122,123,124,125,126,127,128] studies were analyzed for study type, the operation(s) examined, and the risk factors discussed. It is critical to note that all frequency counts in this section represent the number of studies in which each risk factor was examined, reflecting patterns of research attention rather than estimates of biological effect size or causal primacy. The entire list of studies found and used can be seen in Supplementary Table S2.

**Figure 3:**
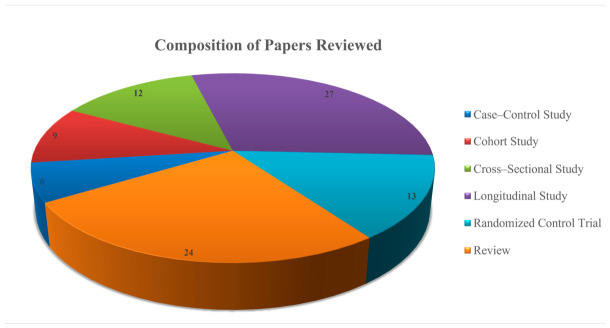
The composition of the papers reviewed. Most papers reviewed belonged in the “Longitudinal Study” category (27). The least reviewed type of paper was the “Case–Control Study” category (6).

The studies were condensed into six main types: Review, Randomized Controlled Trial, Cohort Study, Case–Control Study, Cross-Sectional Study, and Longitudinal Study. Longitudinal studies predominate because disease onset and progression are inherently time-dependent, making repeated-measures designs most appropriate for capturing the dynamic, cumulative nature of risk factor exposure. The relative scarcity of case–control studies (6.6% of included literature) has meaningful implications: case–control designs are particularly powerful for identifying novel or rare risk factors and estimating odds ratios, and their underrepresentation limits the literature’s capacity to generate precise, adjusted effect size estimates for individual BRD risk factors. This is a critical gap warranting targeted investment in future research. Articles and meta-analyses were included under the “review” category. Retrospective analyses were included under the “longitudinal study” category. Surveys were included under the “cross-sectional study.” The composition of the total papers reviewed is shown in Figure 3.

**Figure 4:**
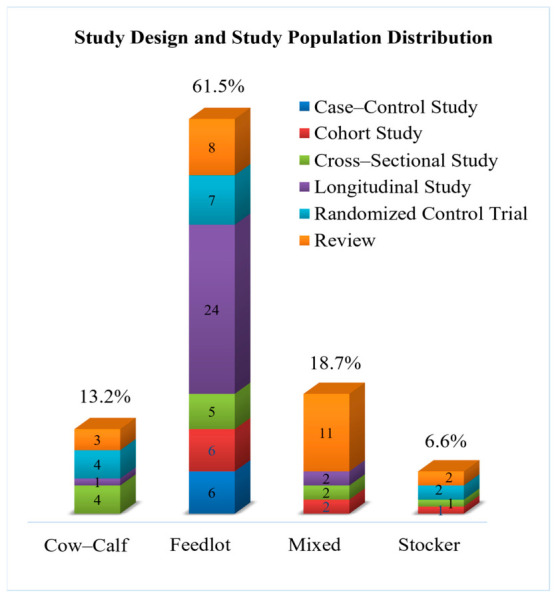
A depiction of how many of each study category studied a specific operation in the beef production industry. Most studies focused on feedlot operations, while the least studied were stocker operations.

For each category, the number of studies was listed. Next, each category was split into how many studies discussed a certain beef production stage: cow–calf, stocker, feedlot, and mixed (papers discussing multiple beef production systems) (Figure 4). The specific risk factors addressed by all papers for a certain operation were then listed (Supplementary Table S3). Most of the studies analyzed were either reviews or longitudinal studies. The most analyzed operation was the feedlot (62%), with most being longitudinal studies. Stocker operations were discussed the least (7%). Oftentimes, researchers prioritize feedlot studies due to their measurable outcomes in feed efficiency, carcass quality, and greenhouse gas emissions, which are critical for both industry competitiveness and policy development [129]. This is unlike cow–calf and stocker systems, which are more dispersed, variable, and less directly tied to market endpoints, making them more challenging to study at scale.

**Figure 5:**
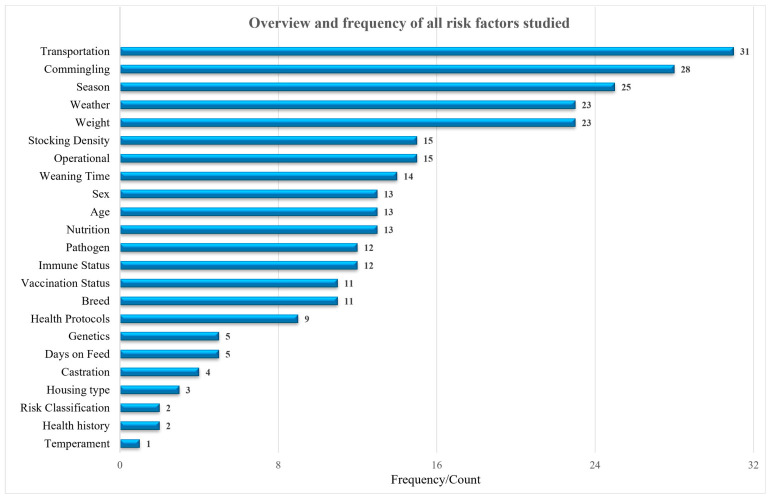
The 23 key risk factors for BRD were identified, extracted, and categorized based on their varying research frequencies in the included research articles.

**Figure 6:**
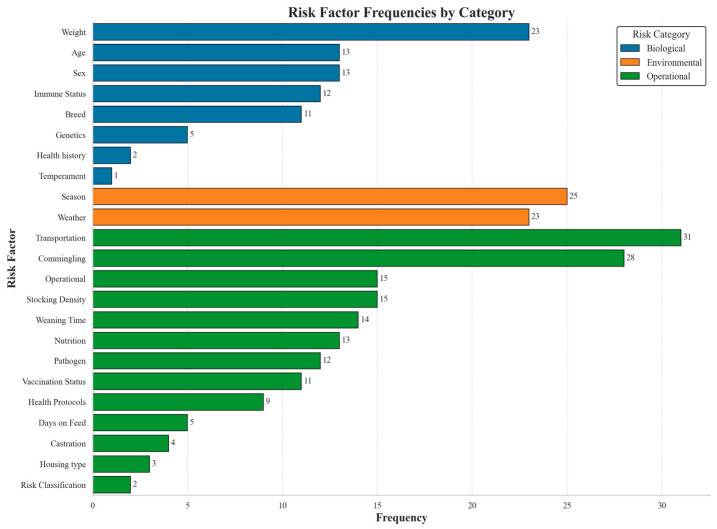
A bar chart categorizing risk factors across the three risk domains (biological, operational, and environmental) and the specific frequencies of the risk factors. For clarity: weight is classified in the biological domain; season is classified in the environmental domain; and transportation is classified in the operational domain.

**Figure 7:**
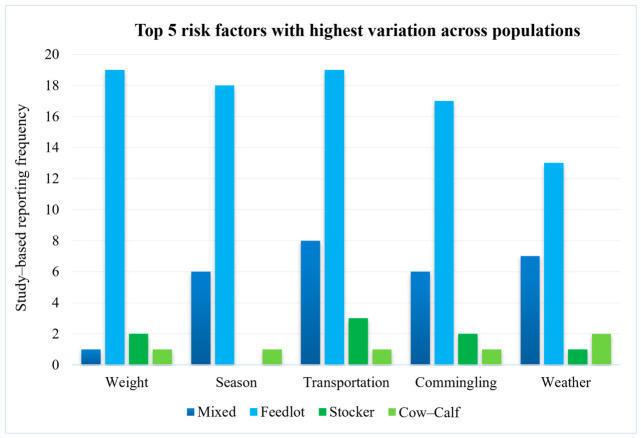
The top five risk factors with the highest variation across beef production stages based on their research frequency in the reviewed articles.

**Figure 8:**
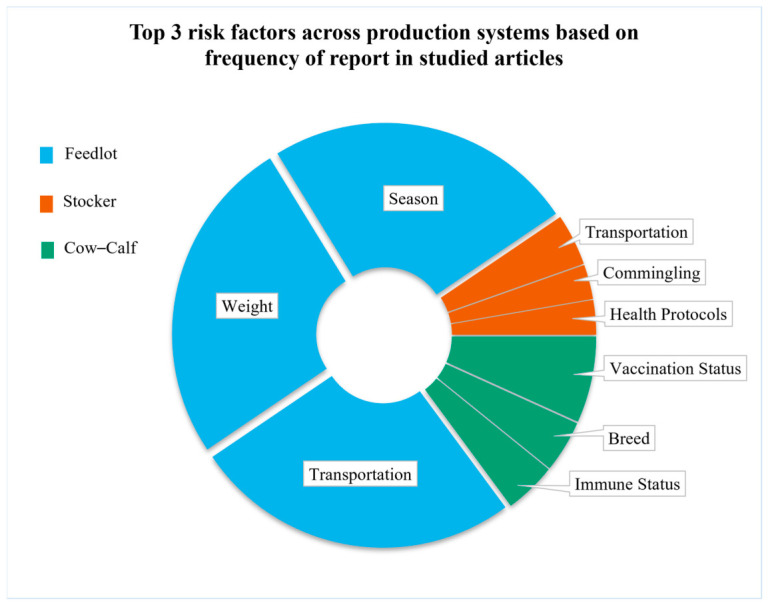
Figure showing top three risk factors across production systems. The top three risk factors in cow–calf operations are vaccination status, immune status, and breed. The top three risk factors for stocker operations are transportation, health protocols, and commingling. The top three risk factors in feedlot operations are transportation, weight, and season.

From the studies, risk factors for BRD occurrence across all operations were identified and compiled into a graph (Figure 5). Notice that the frequencies in the following figures (Figure 5, Figure 6, Figure 7 and Figure 8) represent the number of studies in which each risk factor was examined, reflecting research attention rather than estimates of biological effect size or relative risk. For each of the 23 risk factors, high-risk state definitions are provided in Table A1 (See Appendix A). Transportation was the most frequently examined risk factor across the included studies, followed by commingling.

Research emphasis on transportation demonstrates that it is a key operational determinant of BRD risk. Longer transport durations—six hours or more—were associated with increased BRD occurrence [21], a finding reinforced by evidence linking prolonged transport with elevated physiological stress and consequent increases in disease morbidity [130]. An emerging microbiome-related pathway may further explain this association: rest-stop addition during transportation produced compositional shifts in the nasopharyngeal microbiota [88], though these changes were not yet definitively linked to disease outcome. A related review [61] highlights that disruption of the respiratory tract microbiota may facilitate colonization by opportunistic pathogens; whether transportation induces comparable dysbiosis remains a hypothesis warranting further mechanistic investigation.

Moreover, commingling is also a highly relevant BRD risk factor. The timing and management context of commingling substantially modulate its effect on BRD risk [69]. Cattle not pre-acclimated with animals from different herds for at least 12 days before pen introduction were at higher risk than those mixed at least four weeks before introduction, suggesting that gradual social pre-adaptation reduces transmission risk at feedlot entry [69]. Herd size interacts with commingling: groups exceeding 50 cattle had lower BRD risk when the group had been established for at least 13 days before introduction. In contrast, a study on nursing beef calves found that larger herd sizes were positively associated with BRD detection [48], possibly reflecting increased opportunities for pathogen transmission or greater clinical surveillance capacity in larger groups. Ref. [69] cautions that many herd-size studies fail to report the duration of group establishment, limiting direct comparisons across studies. Beyond grouping timing and social hierarchy, additional effect modifiers of the commingling–BRD relationship include age variation within commingled groups as a determinant of immune competence heterogeneity, the number of source origins as a modulator of pathogen diversity and infection pressure, and pen ventilation as an environmental co-factor that interacts with stocking density to amplify or attenuate airborne transmission risk [14,61,69].

### 4.2. Risk Factor Classification

We organized risk factors into biological, operational, and environmental categories (Figure 6). Operational risk factors included those resulting from normal day-to-day operational procedures. Commingling and transportation fall under this category, as well as vaccination, weaning time, castration, risk classification, and other management procedures. Biological risk factors are those inherent to the cattle. Genetics, age, weight, sex, temperament, and nutrition are included under this classification. In addition, the type of pathogen, stress, and immune status fall under this category. Environmental factors are essentially weather conditions and seasonality, which cannot be totally controlled. The most discussed factors affecting BRD in beef cattle production were operational, specifically, commingling and transportation. Biological and environmental factors were evenly discussed. Under biological factors, weight was most addressed. Under environmental factors, season and weather were discussed equally.

In addition to operational and host-related risk factors, the included studies consistently reported viral and bacterial pathogens associated with BRD, including BRSV, BVDV, BCoV, and BHV-1, as well as *M. haemolytica*, *P. multocida*, and *M. bovis* [77,78,83,86,115]. Co-infections and polymicrobial disease states were frequently described, consistent with the established model in which viral priming and concurrent management stressors predispose cattle to secondary bacterial pneumonia [2]. Pathogen emphasis varies by region. Studies from Australia more frequently emphasized viral pathogens and their interaction with transport stressors [16,36,60,61,62,108]. This pattern is consistent with the influence of national eradication and control programs that may alter bacterial pathogen prevalence and detection patterns, rather than representing a definitive conclusion that viral pathogens are more biologically important in Australia. In contrast, studies from North America and Europe have more commonly identified *M. haemolytica, P. multocida, and M. bovis* as dominant bacterial contributors [9,57,81,83], often following viral priming events [2]. These regional differences highlight how surveillance priorities and production system characteristics shape pathogen profiles and warrant region-specific interpretation of BRD epidemiology.

### 4.3. Risk Factors Across Production Systems

Once the risk factors were identified and classified, they were examined for their frequency of occurrence and how they vary within each stage of beef production. Figure 7 shows this result. The top five predictors of BRD with the greatest variation across populations were commingling, season, transportation, weather, and weight. All five were significantly represented in feedlot-related studies when compared to cow–calf, stocker, and mixed operations. This may be linked to the fact that more than 50% of the total studies considered in our review were focused on feedlot operations. Identifying which specific risk factors are more common in each operation of integrated beef production is much harder to pinpoint. Most factors have some level of significance at each stage.

#### 4.3.1. Cow–Calf Production System

Of the selected papers on cow–calf operations, many focused on operational and biological factors. Vaccination status and timing were important in reducing BRD risk in cow–calf production (Figure 8). For instance, ref. [62] emphasized the importance of implementing efficient vaccination programs. These programs would ideally begin before calving season and include management of other factors such as nutrition, pen environment, weaning, and more. However, another review emphasized the limited number of studies on such factors in cow–calf operations [51]. The review focused on transportation risks but highlighted that most studies focusing on management, vaccination, metaphylaxis, and nutrition were related to feedlot operations. Being able to translate these studies to the cow–calf stage would enable better analyses of cow–calf risk factors. Understanding whether the same management strategies used in the feedlot stage could be applied in the cow–calf stage would expand the understanding of BRD in cow–calf populations.

#### 4.3.2. Stocker Production System

Calves entering the stocker phase are typically not ready for the feedlot market [61]. Post-weaning risk factors, including transportation, commingling, inclement weather, and increased pathogen pressure, influence calf health outcomes [73] as calves enter the stocker phase. Proper management programs, documentation of incoming cattle health, and adequate nutritional care could reduce BRD risk at this stage [131].

#### 4.3.3. Feedlot Production System

Based on this review’s findings, the feedlot stage seems to have the most risk factors studied. The biological, operational, and environmental risk factors all play a role when examining the occurrence of BRD in the feedlot [5,41,67,129]. However, the most frequently reported risk factors in the feedlot production system across the articles studied are transportation, weight, and season (Figure 8). It is worth mentioning that the administration of antimicrobials upon arrival was commonly reported in our review. Metaphylaxis, for instance, is often used once cattle reach the feedlot stage, which reduces the risk of BRD-associated pathogens colonizing the respiratory tract [105]. However, this practice is uncommon in the cow–calf stage [51]. Perhaps bolstering cattle’s immune status (i.e., vaccination) and lowering the burden of infection (i.e., antibiotic use) are the most prevalent ways to reduce BRD risk in feedlot cattle, as opposed to purely managemental methods.

#### 4.3.4. Interaction of Risk Factors

The results of this review highlight the importance of understanding how environmental, biological, and operational risk factors interact and affect BRD risk. No single risk factor can be attributed to BRD; instead, the risk stems from multiple factors affecting cattle and one another (risk interaction). A key explanatory concept is the “BRD risk threshold”: multiple sub-threshold stressors, when concurrent, can collectively exceed the threshold for disease initiation. Ref. [132] documented that the co-occurrence of commingling, transportation, and weaning stress in stocker cattle was associated with substantially higher BRD morbidity rates than any single factor alone. Ref. [133] provide immunological evidence of additive or synergistic immune suppression under concurrent stressor exposure. Ref. [71] modeled the combined effects of metaphylaxis and pen size on BRD outcomes, while ref. [34] examined multi-stressor farming practice interactions. Where specific numeric interaction estimates were unavailable in the reviewed literature, this gap is explicitly acknowledged as a critical priority for future investigation.

## 5. Limitations of the Current Study

Although this review was conducted in accordance with PRISMA-ScR guidelines to ensure breadth and methodological rigor, several limitations warrant consideration [52]. First, the geographic and linguistic restrictions narrowed the scope of evidence. This exclusion criterion introduces systematic geographic bias: persistent high ambient temperatures and humidity in tropical and subtropical regions may independently elevate respiratory stress and alter pathogen persistence; pathogen profiles in tropical beef systems may differ substantially (e.g., greater prevalence of parasitic and fungal co-infections); and herd management in tropical production systems may involve risk factors largely absent from temperate intensive systems. Consequently, the conclusions of this review should be understood as applicable primarily to temperate intensive beef production systems in North America, Australia, and Europe. This may not generalize to tropical or extensive production contexts.

A second limitation is the predominance of feedlot-focused studies. Many identified risk factors are disproportionately characterized in feedlot contexts [5,14] while early-life determinants, including colostrum management, pre-weaning nutrition, and housing conditions in cow–calf systems, remain underrepresented [51]. This stage-specific bias constrains the review’s ability to develop integrative models that account for cumulative and interacting risk factors across the full beef production continuum.

Publication bias is an additional limitation: studies reporting positive or significant associations are more likely to be published than null-result studies, potentially inflating the apparent importance of certain risk factors. The equal weighting of review articles and primary studies in frequency counts is consistent with scoping review methodology [52,53,134], but results should be interpreted with this design decision in mind.

Methodological heterogeneity further complicates synthesis. Studies employing strict laboratory-confirmed case definitions may systematically report different risk factor profiles than those relying on clinical scoring alone [22,23]. Studies using clinical scoring may emphasize more readily observable operational risk factors (transportation, commingling), whereas studies using pathogen-specific diagnostics may better capture biological and environmental drivers. It is therefore possible that the frequency distribution of risk factors in this review partly reflects the predominance of clinical scoring-based study designs rather than the true biological distribution of risk.

Finally, formal inter-rater reliability statistics (e.g., Cohen’s kappa) was not calculated for reviewer agreement, and the scarcity of multi-omics datasets reduces the capacity to model dynamic, cumulative risk across production stages. These constraints underscore the need for standardized diagnostic frameworks, harmonized data collection protocols, and integrative omics-enabled analytical approaches.

## 6. Future Research Directions and Implications

These findings lay the groundwork for future developments in data-driven decision-making in integrated beef production systems. Identifying the key risk factors contributing to BRD could open up new avenues for BRD management. Based on the gaps identified in this review, three specific and actionable research proposals are advanced:

Longitudinal, cross-stage cohort studies: Prospective studies of individual animals from the cow–calf through feedlot stage are urgently needed to quantify the cumulative and interacting effects of early-life risk factors on BRD susceptibility and identify critical windows for intervention.

Standardized BRD surveillance frameworks: Development and adoption of harmonized, multi-site BRD case definitions and data-collection protocols across North America, Australia, and Europe would enable cross-study meta-analysis and improve detection of stage-specific risk factor patterns.

Integrated risk prediction models incorporating omics data: Future models should integrate genomic, microbiome, and metabolomic data with operational and environmental variables to enable precision, animal-level BRD risk prediction and mitigation, reducing reliance on population-level metaphylactic strategies and supporting antimicrobial stewardship goals.

## 7. Conclusions

Understanding BRD is crucial in beef production, given its significant economic impact and implications for animal welfare. This review underscores the complexity of BRD management, which is primarily influenced by variation in risk factors. The studies highlighted in this review illustrate that BRD is not merely a consequence of infectious agents but is intricately linked to a multitude of factors, including transportation stress, environmental conditions, biological predispositions, and management practices.

Three actionable recommendations emerge from these findings, including prioritization of longitudinal, multi-stage cohort studies to close the cow–calf and stocker evidence gap; development of standardized, cross-regional BRD diagnostic and surveillance frameworks; and integration of antimicrobial stewardship principles, optimized vaccination programs, and precision livestock technologies (AI/ML) into future BRD prevention strategies. These efforts are essential to moving beyond fragmented, stage-specific management toward an integrated, data-driven industry-wide prevention strategy.

## Data Availability

No new data were created or analyzed in this study. Data sharing is not applicable to this article.

## Supplementary Materials

The following supporting information can be downloaded at: https://www.mdpi.com/article/10.3390/ani16111726/s1, Table S1—PRISMA SCR Checklist; Table S2—Extraction Table; Table S3—Risk Factors by Paper.

## Appendix A

**Table A1:** High-risk state definition of BRD risk factors based on papers studied.

S/N	Risk Factor	Description of High-Risk State
**1**	Transportation	Journeys lasting six hours or more significantly increase stress and morbidity.
**2**	Commingling	Mixing cattle from different origins less than 12 days prior to pen induction.
**3**	Season	Induction season, particularly during fall or periods of peak regional disease prevalence.
**4**	Weather	Significant temperature fluctuations and high humidity that stresses the respiratory tract.
**5**	Weight	Low birth weight in cow–calf systems or light induction weight in feedlots.
**6**	Stocking Density	Overcrowding in pens or confinement that facilitates pathogen spread.
**7**	Operational	Inconsistent husbandry practices or the lack of a unified, multi-sector management strategy.
**8**	Weaning Time	The freshly weaned state, where maternal separation acts as a peak physiological stressor.
**9**	Nutrition	Inadequate maternal nutrition or poor nutritional status upon entering the stocker or feedlot phase.
**10**	Sex	Physiological differences between steers, heifers, and bulls that influence susceptibility.
**11**	Age	Younger cattle due to their developing immune systems and the stress of new environments.
**12**	Immune Status	Failure of passive transfer (inadequate colostrum intake) or general immune suppression.
**13**	Pathogen	Exposure to primary viral infections (e.g., BRSV, BCoV) that predispose the animal to secondary bacterial pneumonia.
**14**	Vaccination Status	Failure to implement efficient programs before calving or weaning.
**15**	Breed	Genetic predisposition; notably, Hereford cattle found to be 10 times more likely to require treatment.
**16**	Health Protocols	Lack of biosecurity protocols at the point of origin or arrival.
**17**	Days on Feed	The initial 45 days post-arrival. This is the highest-risk interval for morbidity.
**18**	Genetics	Inherent susceptibility resulting from a lack of selection for disease-resistance traits.
**19**	Castration	Castration procedures are performed upon arrival at a feedlot rather than earlier in the production cycle.
**20**	Housing Type	Environments with poor ventilation.
**21**	Health History	Incoming cattle with unknown health status or health backgrounds.
**22**	Risk Classification	Cattle categorized as high-risk upon arrival due to a lack of preconditioning or known stressors.
**23**	Temperament	Highly excitable behavior during handling, which triggers stress responses that suppress immunity.
